# (*Z*)-3-Benzyl-2-[(2-phenyl­cyclo­hex-2-en­yl)imino]-1,3-thia­zolidin-4-one

**DOI:** 10.1107/S1600536812033211

**Published:** 2012-07-28

**Authors:** Chin Wei Ooi, Hoong-Kun Fun, Ching Kheng Quah, Murugan Sathishkumar, Alagusundaram Ponnuswamy

**Affiliations:** a X-ray Crystallography Unit, School of Physics, Universiti Sains Malaysia, 11800 USM, Penang, Malaysia; b Department of Organic Chemistry, School of Chemistry, Madurai Kamaraj University, Madurai 625 021, Tamil Nadu, India

## Abstract

The title compound, C_22_H_22_N_2_OS, exists in a *Z* configuration with respect to the N=C bond. The cyclo­hexene ring adopts a distorted sofa conformation. The thia­zolidine ring is essentially planar, with a maximum deviation of 0.030 (2) Å, and forms dihedral angles of 76.66 (6) and 74.55 (6)° with the terminal phenyl rings. The dihedral angle between the phenyl rings is 71.55 (7)°. In the crystal, a C—H⋯π inter­action is observed.

## Related literature

For the bioactivity of thia­zolidin-4-one derivatives, see: Previtera *et al.* (1994 ▶); Sharma *et al.* (2000 ▶); Kato, Ozaki & Tamura (1999 ▶); Kato, Ozaki & Ohi (1999 ▶); Tanabe *et al.* (1991 ▶); Rawal *et al.* (2005 ▶); Voss *et al.* (2003 ▶). For related structures, see: Fun *et al.* (2011 ▶); Ooi *et al.* (2012*a* ▶,*b* ▶,*c* ▶). For ring conformations, see: Cremer & Pople (1975 ▶). For bond-length data, see: Allen *et al.* (1987 ▶). For the stability of the temperature controller used in the data collection, see: Cosier & Glazer (1986 ▶).
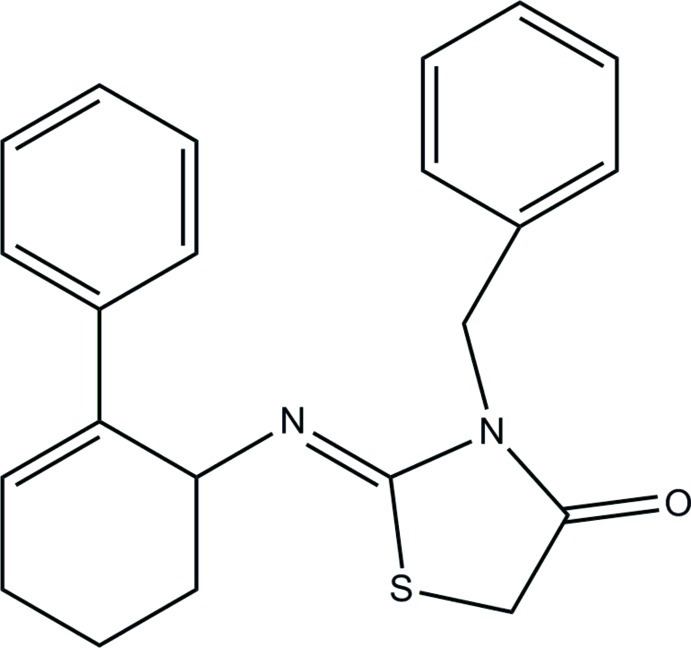


## Experimental

### 

#### Crystal data

C_22_H_22_N_2_OS
*M* *_r_* = 362.48
Monoclinic, 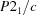

*a* = 12.8400 (2) Å
*b* = 8.9261 (1) Å
*c* = 17.9634 (3) Å
β = 118.476 (1)°
*V* = 1809.72 (5) Å^3^
*Z* = 4
Mo *K*α radiation
μ = 0.19 mm^−1^
*T* = 100 K
0.39 × 0.32 × 0.21 mm

#### Data collection

Bruker SMART APEXII CCD area-detector diffractometer
Absorption correction: multi-scan (*SADABS*; Bruker, 2009 ▶) *T*_min_ = 0.929, *T*_max_ = 0.960
22263 measured reflections
6670 independent reflections
4979 reflections with *I* > 2σ(*I*)
*R*_int_ = 0.034

#### Refinement

*R*[*F* ^2^ > 2σ(*F* ^2^)] = 0.045
*wR*(*F* ^2^) = 0.115
*S* = 1.03
6670 reflections
235 parameters
H-atom parameters constrained
Δρ_max_ = 0.45 e Å^−3^
Δρ_min_ = −0.29 e Å^−3^

### 

Data collection: *APEX2* (Bruker, 2009 ▶); cell refinement: *SAINT* (Bruker, 2009 ▶); data reduction: *SAINT*; program(s) used to solve structure: *SHELXTL* (Sheldrick, 2008 ▶); program(s) used to refine structure: *SHELXTL* ; molecular graphics: *SHELXTL*; software used to prepare material for publication: *SHELXTL* and *PLATON* (Spek, 2009 ▶).

## Supplementary Material

Crystal structure: contains datablock(s) global, I. DOI: 10.1107/S1600536812033211/is5168sup1.cif

Structure factors: contains datablock(s) I. DOI: 10.1107/S1600536812033211/is5168Isup2.hkl

Supplementary material file. DOI: 10.1107/S1600536812033211/is5168Isup3.cml

Additional supplementary materials:  crystallographic information; 3D view; checkCIF report

## Figures and Tables

**Table 1:** Hydrogen-bond geometry (Å, °) *Cg*1 is the centroid of the C1–C6 phenyl ring.

*D*—H⋯*A*	*D*—H	H⋯*A*	*D*⋯*A*	*D*—H⋯*A*
C22—H22*A*⋯*Cg*1	0.93	2.97	3.7662 (15)	143

**Fig. 1.**
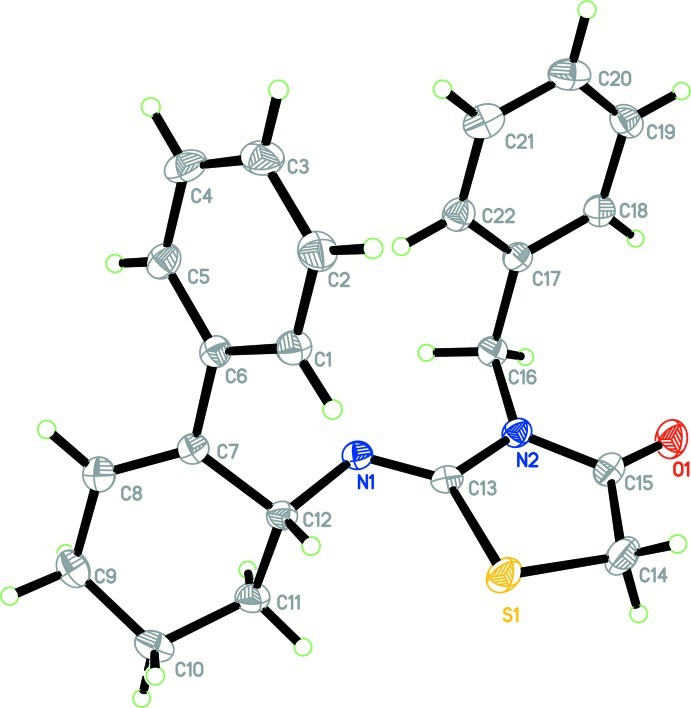
The molecular structure of the title compound, showing 50% probability displacement ellipsoids and the atom-numbering scheme.

**Fig. 2.**
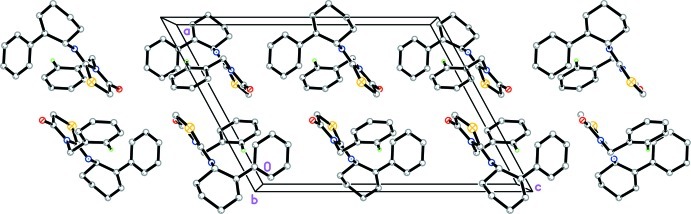
The crystal packing of the title compound.

## supplementary crystallographic information

### Comment

Thiazolidin-4-one derivatives are known to exhibit diverse bioactivities such as anti-histaminic (Previtera *et al.*, 1994), anti-microbial (Sharma *et al.*, 2000; Kato, Ozaki & Tamura, 1999), PAF antagonist (Tanabe *et al.*, 1991), cardioprotective (Kato, Ozaki & Ohi, 1999), anti HIV (Rawal *et al.*, 2005), and tumor necrosis factor-α antagonist activities (Voss *et al.*, 2003).

The title compound (Fig. 1) exists in *cis* configuration with respect to the N1 ═C13 bond [N1 ═C13 = 1.2660 (15) Å]. The cyclohexene (C7–C12) ring adopts a distorted sofa conformation and the puckering parameters are Q = 0.5050 (15) Å, θ = 51.07 (16)° and φ = 201.6 (2)° (Cremer & Pople, 1975). The thiazolidine (S1/N2/C13–C15) ring is essentially planar with a maximum deviation of 0.030 (2) Å at atom C14 and forms dihedral angles of 76.66 (6) and 74.55 (6)°, respectively, with terminal benzene rings (C1–C6 & C17–C22). The dihedral angle between terminal benzene rings is 71.55 (7)°. The bond lengths (Allen *et al.*, 1987) and angles are within normal ranges and are comparable to the related structures (Fun *et al.*, 2011; Ooi *et al.*, 2012*a*,*b*,*c*).

In the crystal packing (Fig. 2), no significant intermolecular hydrogen bond interactions are observed. The crystal is stabilized by C22—H22A···*Cg*1 interactions (Table 1), involving the centroid of the benzene ring (C1–C6; *Cg*1).

### Experimental

A mixture of 1-benzyl-3-(2-phenylcyclohex-2-enyl)thiourea (0.5 g, 2.3 mmol) and chloro acetylchloride (0.35 g, 4.6 mmol) was heated to reflux in 1,4-dioxane (10 ml) at 100°C for 5 h. The reaction mixture was washed with diluted sodium bicarbonate solution (25 ml) and dried over anhydrous sodium sulfate. The solvent was then evaporated under reduced pressure and the resulting residue was subjected to column chromatography using silica gel (60–120 mesh) as the stationary phase and petroleum ether-ethyl acetate (90:10) as the mobile phase to give the pure product. Yield: 0.69 g (83%); *M.p.*: 132–133 °C. Crystals suitable for X-ray study were obtained by recrystallization in dichloromethane.

### Refinement

All H atoms were positioned geometrically (C—H = 0.93, 0.97 and 0.98 Å) and refined using a riding model with *U*_iso_(H) = 1.2*U*_eq_(C). In the final refinement, one outlier (-11 5 25) was omitted.

### Crystal data

**Table:**

C_22_H_22_N_2_OS	*F*(000) = 768
*M**_r_* = 362.48	*D*_x_ = 1.330 Mg m^−^^3^
Monoclinic, *P*2_1_/*c*	Mo *K*α radiation, λ = 0.71073 Å
Hall symbol: -P 2ybc	Cell parameters from 6720 reflections
*a* = 12.8400 (2) Å	θ = 2.6–32.7°
*b* = 8.9261 (1) Å	µ = 0.19 mm^−^^1^
*c* = 17.9634 (3) Å	*T* = 100 K
β = 118.476 (1)°	Block, colourless
*V* = 1809.72 (5) Å^3^	0.39 × 0.32 × 0.21 mm
*Z* = 4	

### Data collection

**Table:**

Bruker SMART APEXII CCD area-detector diffractometer	6670 independent reflections
Radiation source: fine-focus sealed tube	4979 reflections with *I* > 2σ(*I*)
Graphite monochromator	*R*_int_ = 0.034
φ and ω scans	θ_max_ = 32.8°, θ_min_ = 2.3°
Absorption correction: multi-scan (*SADABS*; Bruker, 2009)	*h* = −19→19
*T*_min_ = 0.929, *T*_max_ = 0.960	*k* = −11→13
22263 measured reflections	*l* = −25→27

### Refinement

**Table:**

Refinement on *F*^2^	Primary atom site location: structure-invariant direct methods
Least-squares matrix: full	Secondary atom site location: difference Fourier map
*R*[*F*^2^ > 2σ(*F*^2^)] = 0.045	Hydrogen site location: inferred from neighbouring sites
*wR*(*F*^2^) = 0.115	H-atom parameters constrained
*S* = 1.03	*w* = 1/[σ^2^(*F*_o_^2^) + (0.0474*P*)^2^ + 0.6514*P*] where *P* = (*F*_o_^2^ + 2*F*_c_^2^)/3
6670 reflections	(Δ/σ)_max_ < 0.001
235 parameters	Δρ_max_ = 0.45 e Å^−^^3^
0 restraints	Δρ_min_ = −0.29 e Å^−^^3^

### Special details

**Table:**

Experimental. The crystal was placed in the cold stream of an Oxford Cryosystems Cobra open-flow nitrogen cryostat (Cosier & Glazer, 1986) operating at 100 (1) K.
Geometry. All e.s.d.'s (except the e.s.d. in the dihedral angle between two l.s. planes) are estimated using the full covariance matrix. The cell e.s.d.'s are taken into account individually in the estimation of e.s.d.'s in distances, angles and torsion angles; correlations between e.s.d.'s in cell parameters are only used when they are defined by crystal symmetry. An approximate (isotropic) treatment of cell e.s.d.'s is used for estimating e.s.d.'s involving l.s. planes.
Refinement. Refinement of *F*^2^ against ALL reflections. The weighted *R*-factor *wR* and goodness of fit *S* are based on *F*^2^, conventional *R*-factors *R* are based on *F*, with *F* set to zero for negative *F*^2^. The threshold expression of *F*^2^ > σ(*F*^2^) is used only for calculating *R*-factors(gt) *etc*. and is not relevant to the choice of reflections for refinement. *R*-factors based on *F*^2^ are statistically about twice as large as those based on *F*, and *R*- factors based on ALL data will be even larger.

### Fractional atomic coordinates and isotropic or equivalent isotropic displacement parameters (Å^2^)

**Table:**

	*x*	*y*	*z*	*U*_iso_*/*U*_eq_	
S1	0.36793 (3)	0.30018 (4)	0.40002 (2)	0.02412 (8)	
O1	0.40661 (8)	0.70672 (11)	0.34194 (6)	0.0271 (2)	
N1	0.16966 (8)	0.40320 (11)	0.40509 (6)	0.01676 (19)	
N2	0.28594 (8)	0.57353 (12)	0.37911 (6)	0.01740 (19)	
C1	0.27121 (10)	0.31490 (14)	0.59927 (8)	0.0202 (2)	
H1A	0.3136	0.2657	0.5767	0.024*	
C2	0.33166 (11)	0.38392 (16)	0.67770 (8)	0.0246 (3)	
H2A	0.4139	0.3781	0.7078	0.030*	
C3	0.27033 (12)	0.46163 (16)	0.71150 (8)	0.0260 (3)	
H3A	0.3110	0.5091	0.7637	0.031*	
C4	0.14737 (12)	0.46752 (16)	0.66629 (8)	0.0251 (3)	
H4A	0.1056	0.5201	0.6883	0.030*	
C5	0.08622 (11)	0.39566 (15)	0.58865 (8)	0.0210 (2)	
H5A	0.0039	0.3991	0.5597	0.025*	
C6	0.14702 (10)	0.31810 (13)	0.55349 (7)	0.0171 (2)	
C7	0.08369 (9)	0.24054 (14)	0.47038 (7)	0.0168 (2)	
C8	−0.01735 (10)	0.16544 (14)	0.44784 (8)	0.0204 (2)	
H8A	−0.0494	0.1678	0.4846	0.025*	
C9	−0.08255 (10)	0.07754 (15)	0.36700 (8)	0.0238 (3)	
H9A	−0.1500	0.1351	0.3266	0.029*	
H9B	−0.1121	−0.0149	0.3784	0.029*	
C10	−0.00165 (11)	0.04137 (15)	0.32905 (8)	0.0238 (3)	
H10A	0.0564	−0.0330	0.3636	0.029*	
H10B	−0.0479	0.0007	0.2725	0.029*	
C11	0.06081 (11)	0.18384 (15)	0.32531 (8)	0.0215 (2)	
H11A	0.0021	0.2586	0.2922	0.026*	
H11B	0.1076	0.1627	0.2972	0.026*	
C12	0.14166 (10)	0.24639 (14)	0.41394 (7)	0.0170 (2)	
H12A	0.2152	0.1882	0.4402	0.020*	
C13	0.25958 (9)	0.42884 (13)	0.39558 (7)	0.0165 (2)	
C14	0.43819 (12)	0.44070 (16)	0.36673 (10)	0.0288 (3)	
H14A	0.5217	0.4477	0.4074	0.035*	
H14B	0.4309	0.4142	0.3121	0.035*	
C15	0.37776 (10)	0.58888 (15)	0.36075 (8)	0.0212 (2)	
C16	0.21796 (10)	0.70455 (14)	0.38096 (7)	0.0183 (2)	
H16A	0.1399	0.6726	0.3705	0.022*	
H16B	0.2079	0.7734	0.3362	0.022*	
C17	0.27962 (9)	0.78435 (13)	0.46541 (7)	0.0172 (2)	
C18	0.34360 (10)	0.91455 (14)	0.47333 (8)	0.0197 (2)	
H18A	0.3475	0.9524	0.4265	0.024*	
C19	0.40185 (10)	0.98879 (15)	0.55063 (8)	0.0226 (2)	
H19A	0.4447	1.0756	0.5555	0.027*	
C20	0.39554 (11)	0.93244 (16)	0.62022 (8)	0.0248 (3)	
H20A	0.4332	0.9827	0.6717	0.030*	
C21	0.33340 (11)	0.80141 (16)	0.61358 (8)	0.0249 (3)	
H21A	0.3307	0.7632	0.6608	0.030*	
C22	0.27519 (10)	0.72727 (15)	0.53625 (8)	0.0208 (2)	
H22A	0.2333	0.6397	0.5317	0.025*	

### Atomic displacement parameters (Å^2^)

**Table:**

	*U*^11^	*U*^22^	*U*^33^	*U*^12^	*U*^13^	*U*^23^
S1	0.02555 (14)	0.01899 (15)	0.03463 (18)	0.00580 (11)	0.01988 (13)	0.00295 (13)
O1	0.0295 (4)	0.0247 (5)	0.0331 (5)	−0.0013 (4)	0.0198 (4)	0.0046 (4)
N1	0.0181 (4)	0.0166 (5)	0.0159 (4)	0.0006 (3)	0.0083 (3)	−0.0002 (4)
N2	0.0179 (4)	0.0166 (5)	0.0192 (5)	0.0021 (3)	0.0101 (3)	0.0010 (4)
C1	0.0202 (5)	0.0209 (6)	0.0193 (5)	0.0006 (4)	0.0093 (4)	−0.0007 (5)
C2	0.0244 (5)	0.0275 (7)	0.0197 (6)	−0.0027 (5)	0.0086 (4)	−0.0002 (5)
C3	0.0366 (7)	0.0245 (7)	0.0168 (6)	−0.0034 (5)	0.0127 (5)	−0.0021 (5)
C4	0.0372 (7)	0.0233 (6)	0.0209 (6)	0.0042 (5)	0.0188 (5)	0.0016 (5)
C5	0.0232 (5)	0.0214 (6)	0.0209 (6)	0.0035 (4)	0.0126 (4)	0.0038 (5)
C6	0.0191 (5)	0.0165 (5)	0.0166 (5)	0.0010 (4)	0.0093 (4)	0.0022 (4)
C7	0.0173 (4)	0.0163 (5)	0.0166 (5)	0.0020 (4)	0.0079 (4)	0.0014 (4)
C8	0.0196 (5)	0.0208 (6)	0.0205 (6)	0.0004 (4)	0.0091 (4)	0.0020 (5)
C9	0.0208 (5)	0.0223 (6)	0.0243 (6)	−0.0032 (4)	0.0075 (4)	0.0001 (5)
C10	0.0241 (5)	0.0188 (6)	0.0233 (6)	−0.0003 (4)	0.0070 (4)	−0.0040 (5)
C11	0.0241 (5)	0.0217 (6)	0.0191 (6)	−0.0011 (4)	0.0105 (4)	−0.0033 (5)
C12	0.0178 (4)	0.0157 (5)	0.0181 (5)	0.0009 (4)	0.0090 (4)	−0.0004 (4)
C13	0.0184 (4)	0.0161 (5)	0.0149 (5)	0.0025 (4)	0.0078 (4)	0.0001 (4)
C14	0.0308 (6)	0.0245 (7)	0.0426 (8)	0.0042 (5)	0.0269 (6)	0.0031 (6)
C15	0.0208 (5)	0.0246 (6)	0.0207 (6)	0.0012 (4)	0.0118 (4)	0.0007 (5)
C16	0.0168 (4)	0.0169 (5)	0.0190 (5)	0.0029 (4)	0.0069 (4)	0.0012 (4)
C17	0.0146 (4)	0.0164 (5)	0.0197 (5)	0.0034 (4)	0.0076 (4)	0.0007 (4)
C18	0.0198 (5)	0.0166 (6)	0.0226 (6)	0.0021 (4)	0.0101 (4)	0.0017 (4)
C19	0.0186 (5)	0.0181 (6)	0.0279 (6)	0.0007 (4)	0.0084 (4)	−0.0023 (5)
C20	0.0231 (5)	0.0249 (7)	0.0204 (6)	0.0067 (5)	0.0055 (4)	−0.0032 (5)
C21	0.0279 (6)	0.0257 (7)	0.0215 (6)	0.0073 (5)	0.0122 (5)	0.0034 (5)
C22	0.0208 (5)	0.0197 (6)	0.0234 (6)	0.0023 (4)	0.0118 (4)	0.0014 (5)

### Geometric parameters (Å, º)

**Table:**

S1—C13	1.7762 (12)	C9—H9B	0.9700
S1—C14	1.8054 (14)	C10—C11	1.5220 (18)
O1—C15	1.2153 (16)	C10—H10A	0.9700
N1—C13	1.2660 (15)	C10—H10B	0.9700
N1—C12	1.4724 (16)	C11—C12	1.5323 (16)
N2—C15	1.3742 (15)	C11—H11A	0.9700
N2—C13	1.4020 (16)	C11—H11B	0.9700
N2—C16	1.4691 (15)	C12—H12A	0.9800
C1—C2	1.3872 (17)	C14—C15	1.5112 (18)
C1—C6	1.4033 (16)	C14—H14A	0.9700
C1—H1A	0.9300	C14—H14B	0.9700
C2—C3	1.388 (2)	C16—C17	1.5133 (17)
C2—H2A	0.9300	C16—H16A	0.9700
C3—C4	1.3900 (19)	C16—H16B	0.9700
C3—H3A	0.9300	C17—C18	1.3909 (17)
C4—C5	1.3888 (18)	C17—C22	1.3968 (18)
C4—H4A	0.9300	C18—C19	1.3915 (18)
C5—C6	1.3992 (17)	C18—H18A	0.9300
C5—H5A	0.9300	C19—C20	1.385 (2)
C6—C7	1.4873 (16)	C19—H19A	0.9300
C7—C8	1.3401 (16)	C20—C21	1.389 (2)
C7—C12	1.5178 (17)	C20—H20A	0.9300
C8—C9	1.5053 (18)	C21—C22	1.3915 (18)
C8—H8A	0.9300	C21—H21A	0.9300
C9—C10	1.5241 (19)	C22—H22A	0.9300
C9—H9A	0.9700		
			
C13—S1—C14	92.31 (6)	C12—C11—H11B	109.3
C13—N1—C12	118.15 (10)	H11A—C11—H11B	108.0
C15—N2—C13	117.62 (10)	N1—C12—C7	109.12 (10)
C15—N2—C16	120.86 (10)	N1—C12—C11	108.27 (10)
C13—N2—C16	121.51 (9)	C7—C12—C11	112.11 (9)
C2—C1—C6	121.02 (12)	N1—C12—H12A	109.1
C2—C1—H1A	119.5	C7—C12—H12A	109.1
C6—C1—H1A	119.5	C11—C12—H12A	109.1
C1—C2—C3	120.45 (12)	N1—C13—N2	121.50 (10)
C1—C2—H2A	119.8	N1—C13—S1	128.48 (10)
C3—C2—H2A	119.8	N2—C13—S1	110.02 (8)
C2—C3—C4	119.12 (12)	C15—C14—S1	108.11 (9)
C2—C3—H3A	120.4	C15—C14—H14A	110.1
C4—C3—H3A	120.4	S1—C14—H14A	110.1
C5—C4—C3	120.67 (12)	C15—C14—H14B	110.1
C5—C4—H4A	119.7	S1—C14—H14B	110.1
C3—C4—H4A	119.7	H14A—C14—H14B	108.4
C4—C5—C6	120.79 (11)	O1—C15—N2	124.27 (12)
C4—C5—H5A	119.6	O1—C15—C14	124.27 (11)
C6—C5—H5A	119.6	N2—C15—C14	111.46 (11)
C5—C6—C1	117.92 (11)	N2—C16—C17	111.40 (9)
C5—C6—C7	121.86 (10)	N2—C16—H16A	109.3
C1—C6—C7	120.22 (11)	C17—C16—H16A	109.3
C8—C7—C6	121.55 (11)	N2—C16—H16B	109.3
C8—C7—C12	121.86 (11)	C17—C16—H16B	109.3
C6—C7—C12	116.55 (10)	H16A—C16—H16B	108.0
C7—C8—C9	124.42 (12)	C18—C17—C22	119.36 (11)
C7—C8—H8A	117.8	C18—C17—C16	119.80 (11)
C9—C8—H8A	117.8	C22—C17—C16	120.83 (11)
C8—C9—C10	110.96 (10)	C17—C18—C19	120.68 (12)
C8—C9—H9A	109.4	C17—C18—H18A	119.7
C10—C9—H9A	109.4	C19—C18—H18A	119.7
C8—C9—H9B	109.4	C20—C19—C18	119.52 (12)
C10—C9—H9B	109.4	C20—C19—H19A	120.2
H9A—C9—H9B	108.0	C18—C19—H19A	120.2
C11—C10—C9	109.18 (11)	C19—C20—C21	120.45 (12)
C11—C10—H10A	109.8	C19—C20—H20A	119.8
C9—C10—H10A	109.8	C21—C20—H20A	119.8
C11—C10—H10B	109.8	C20—C21—C22	119.96 (13)
C9—C10—H10B	109.8	C20—C21—H21A	120.0
H10A—C10—H10B	108.3	C22—C21—H21A	120.0
C10—C11—C12	111.64 (10)	C21—C22—C17	120.02 (12)
C10—C11—H11A	109.3	C21—C22—H22A	120.0
C12—C11—H11A	109.3	C17—C22—H22A	120.0
C10—C11—H11B	109.3		
			
C6—C1—C2—C3	−1.9 (2)	C12—N1—C13—S1	−5.83 (16)
C1—C2—C3—C4	1.0 (2)	C15—N2—C13—N1	−173.23 (11)
C2—C3—C4—C5	0.6 (2)	C16—N2—C13—N1	6.54 (17)
C3—C4—C5—C6	−1.2 (2)	C15—N2—C13—S1	6.76 (13)
C4—C5—C6—C1	0.25 (19)	C16—N2—C13—S1	−173.47 (8)
C4—C5—C6—C7	−179.85 (12)	C14—S1—C13—N1	173.49 (12)
C2—C1—C6—C5	1.27 (19)	C14—S1—C13—N2	−6.49 (9)
C2—C1—C6—C7	−178.63 (12)	C13—S1—C14—C15	4.84 (10)
C5—C6—C7—C8	−39.26 (18)	C13—N2—C15—O1	176.88 (12)
C1—C6—C7—C8	140.65 (13)	C16—N2—C15—O1	−2.89 (18)
C5—C6—C7—C12	142.81 (12)	C13—N2—C15—C14	−3.02 (15)
C1—C6—C7—C12	−37.29 (16)	C16—N2—C15—C14	177.21 (11)
C6—C7—C8—C9	−176.52 (11)	S1—C14—C15—O1	178.04 (11)
C12—C7—C8—C9	1.30 (19)	S1—C14—C15—N2	−2.07 (14)
C7—C8—C9—C10	18.81 (18)	C15—N2—C16—C17	−83.38 (13)
C8—C9—C10—C11	−49.49 (14)	C13—N2—C16—C17	96.86 (12)
C9—C10—C11—C12	63.30 (13)	N2—C16—C17—C18	101.26 (12)
C13—N1—C12—C7	148.59 (10)	N2—C16—C17—C22	−77.43 (13)
C13—N1—C12—C11	−89.14 (12)	C22—C17—C18—C19	−0.68 (17)
C8—C7—C12—N1	130.49 (12)	C16—C17—C18—C19	−179.39 (10)
C6—C7—C12—N1	−51.58 (13)	C17—C18—C19—C20	−0.22 (18)
C8—C7—C12—C11	10.56 (16)	C18—C19—C20—C21	1.14 (18)
C6—C7—C12—C11	−171.51 (10)	C19—C20—C21—C22	−1.14 (18)
C10—C11—C12—N1	−163.06 (10)	C20—C21—C22—C17	0.22 (18)
C10—C11—C12—C7	−42.63 (14)	C18—C17—C22—C21	0.68 (17)
C12—N1—C13—N2	174.16 (10)	C16—C17—C22—C21	179.37 (10)

### Hydrogen-bond geometry (Å, º)

*Cg*1 is the centroid of the C1–C6 phenyl ring.

**Table:**

*D*—H···*A*	*D*—H	H···*A*	*D*···*A*	*D*—H···*A*
C22—H22*A*···*Cg*1	0.93	2.97	3.7662 (15)	143
